# Dynamic Data Structures for Timed Automata Acceptance

**DOI:** 10.1007/s00453-022-01025-8

**Published:** 2022-09-05

**Authors:** Alejandro Grez, Filip Mazowiecki, Michał Pilipczuk, Gabriele Puppis, Cristian Riveros

**Affiliations:** 1 Pontificia Universidad Católica de Chile, Santiago, Chile; 2 Millennium Institute for Foundational Research on Data, Santiago, Chile; 3 University of Warsaw, Warsaw, Poland; 4 University of Udine, Udine, Italy

**Keywords:** Timed automata, Data stream, Dynamic data structure, Theory of computation, Models of computation

## Abstract

We study a variant of the classical membership problem in automata theory, which consists of deciding whether a given input word is accepted by a given automaton. We do so through the lenses of parameterized dynamic data structures: we assume that the automaton is fixed and its size is the parameter, while the input word is revealed as in a stream, one symbol at a time following the natural order on positions. The goal is to design a dynamic data structure that can be efficiently updated upon revealing the next symbol, while maintaining the answer to the query on whether the word consisting of symbols revealed so far is accepted by the automaton. We provide complexity bounds for this dynamic acceptance problem for timed automata that process symbols interleaved with time spans. The main contribution is a dynamic data structure that maintains acceptance of a fixed one-clock timed automaton ${\mathcal {A}}$ with amortized update time $2^{{\mathcal {O}}(|{\mathcal {A}}|)}$ per input symbol.

## Introduction

Imagine we would like to monitor whether the behaviour of a server is correct. The run of the server can be abstracted by an infinite stream $w=a_1 a_2 a_3\ldots \in \Sigma ^\omega$, where $\Sigma$ is a finite alphabet of possible events. The events are disclosed one at a time on the input, and at every moment we should tell whether the prefix consisting of the events observed so far is correct.

**Fig. 1:**
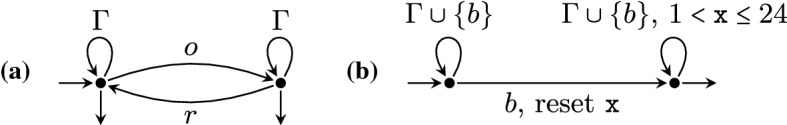
Left: a finite automaton ${\mathcal {A}}$ recognising language $\Gamma ^*(o\Gamma ^*r\Gamma ^*)^*(\{\varepsilon \} \cup o\Gamma ^*)$, where occurrences of *o* are interleaved by occurrences of *r*. Right: a timed automaton ${\mathcal {B}}$ with single clock $\mathtt {x}$

A simple yet expressive formalism for describing properties of such *data streams* is provided by classical finite automata. For example, suppose we would like to verify the property that a certain resource is being used by at most one process. Assume that the alphabet is $\Sigma = \{o,r\} \cup \Gamma$, where *o* denotes a request of the resource, *r* denotes a release of the resource, and $\Gamma$ contains other immaterial events. The streams satisfying the discussed property can be then characterised as those where every prefix is accepted by the two-state automaton ${\mathcal {A}}$ of Fig. 1. Here, a state indicates whether the resource is currently available or not.

Verifying the correctness of a stream over time can be formalized through the following *dynamic acceptance problem*: for a fixed automaton ${\mathcal {A}}$, design a *data structure* that upon receiving subsequent events from the stream, monitors whether the prefix read so far is accepted by ${\mathcal {A}}$. An obvious, though usually suboptimal solution would be to store in the data structure the prefix read so far, and, upon receiving a new symbol, run the automaton on the whole prefix. This would require time linear in the total length of the prefix, which after a while can become very large compared to $|{\mathcal {A}}|$, the size of the automaton ${\mathcal {A}}$. So we would like to minimize the update time by smartly organizing and reusing information computed before.

Cast in this way, the dynamic acceptance problem naturally lends itself to a treatment using the notions of parametrised complexity. Namely, we consider the automaton ${\mathcal {A}}$ fixed and use the parameter $|{\mathcal {A}}|$ as a measure for expressing guarantees on the update time. Ideally, we would like to obtain update time bounded by a computable function of $|{\mathcal {A}}|$ only. This way, our work inscribes into the area of *parameterized dynamic data structures*, which is a direction that is still relatively unexplored, but starts to attract considerable attention; see *e.g.*, [1–3] and references therein for an overview of recent advances.

For finite automata, the dynamic acceptance problem can be solved easily with update time ${\mathcal {O}}(|{\mathcal {A}}|)$, as follows. After reading a prefix *u*, the data structure stores the subset of states $S\subseteq Q$ in which the automaton may be after reading *u* (in general, we allow the automaton to be non-deterministic). Upon receiving the next input symbol, the set *S* is updated by applying the possible transitions on every state in *S*. Moreover, telling whether ${\mathcal {A}}$ accepts the current input prefix boils down to checking whether *S* contains an accepting state. Both the update and the query described above can be implemented in time linear in $|{\mathcal {A}}|$.

Unfortunately, real-life scenarios involve many aspects that cannot be captured by a simple formalism such as finite automata. One of these aspects is *time*. Consider the following example of property that needs to be verified: at every moment in time when an event occurs, a special backup operation has been performed within the last 24 hours, but not within the last 1 hour. A natural choice to model this and similar properties is to enhance finite automata with the ability of measuring time, by adding one or more *clocks* and allowing transitions with constraints on clocks – a formal definition of this automaton model, called *timed automaton*, will be given in Sect. 2. The above property could be verified at every incoming event by testing whether the sequence of events received is accepted by a suitable timed automaton. The timed automaton would have one clock $\mathtt {x}$ and two states, “before backup” and “after backup”, and would behave as follows (see the right hand-side of Fig. 1). More precisely, for every incoming event, the timed automaton non-deterministically guesses a backup event *b* and verifies that it occurred within the last 24 hours, but not within the last 1 hour. Thus, upon reading an occurrence of a backup event *b*, the timed automaton may either ignore this event and remain in the initial state “before backup”, or move from state “before backup” to state “after backup” while resetting the clock. A sequence *u* of events is accepted if the automaton reaches the final state “after backup” and, for those events that occurred after the reset, the value of the clock is in the range (1, 24].

Timed automata are a central topic in the area of verification, and they have a rich and diverse literature, see *e.g.*, [4–6]. In this work we are interested in the dynamic acceptance problem for timed automata, defined analogously to that for finite automata.

Note that in the setting of timed automata, the same technique that worked for finite automata will not work so easily. The reason is that for a finite automaton ${\mathcal {A}}$, the set of configurations in which ${\mathcal {A}}$ may be is a subset of the set of control states, whose size is bounded by the size of ${\mathcal {A}}$. On the other hand, a configuration of a timed automaton consists of a control state and a tuple of clock values, so the number of possible configurations is a priori unbounded. Concretely, after reading a prefix of length *n*, there may be as many as ${\mathcal {O}}(|{\mathcal {A}}|\cdot n^k)$ different configurations which the given *k*-clock timed automaton may possibly reach, due to non-determinism and clock resets. Efficient maintenance of this configuration set in a data structure poses the main conceptual challenge in this paper.

*Our contribution* We design a dynamic data structure that, for a fixed timed automaton ${\mathcal {A}}$ with *one* clock, monitors whether ${\mathcal {A}}$ accepts the prefix read so far with amortized update time ${\mathcal {O}}(2^{3|{\mathcal {A}}|})$. This can be improved to worst-case (*i.e.*, non-amortized) update time when the input stream is *discrete*, that is, when all time spans between consecutive events are equal. Our data structure actually works in a slightly more general setting, where the automaton ${\mathcal {A}}$ is not entirely fixed, but rather is provided on input upon initialization of the data structure.

We also give a somewhat complementary lower bound: under the 3SUM Conjecture, we prove that there exists a fixed timed automaton ${\mathcal {A}}$ with two clocks and additive constraints on them such that no data structure for the dynamic acceptance problem for ${\mathcal {A}}$ may achieve strongly sublinear amortized update time (*i.e.*, time ${\mathcal {O}}(n^{1-\delta })$ for $\delta >0$). Here, by additive constraints we mean that in the transition relation of ${\mathcal {A}}$ we may use affine clock conditions that involve more than one clock, *e.g.*, $\mathtt {x}+\mathtt {y}=c$, where $\mathtt {x},\mathtt {y}$ are clocks and *c* is a constant.

If the given timed automaton ${\mathcal {A}}$ has more than one clock, but only constraints involving a single clock are allowed, it remains open whether there is an efficient data structure for the dynamic acceptance problem or a lower bound similar to the above one.

*Related work* A preliminary version of this work appeared in [7].

The setting is close to *runtime verification* [8], an area that focuses on verification techniques that could be performed at runtime, e.g. using timed automata [9, 10]. However, while we study monitoring a data stream through a suitable data structure in the *dynamic* setting, studies on runtime verification typically focus on *static* problems. An example of such a problem is: given an input prefix *u*, verify whether there is a sequence of events that extends *u* to a word accepted by the device (*e.g.*, a finite automaton). The problem studied in [11] is similar to the setting presented here; however, this line of work considers clock constants (*e.g.*, 24 in Fig. 1) as part of the input, contributing to the parameter of the complexity bounds, and this considerably simplifies the setting (see Sects. 2 and 3).

The dynamic acceptance problem that we consider here resembles the setting of *streaming algorithms*; see *e.g.*, [12–14] for works with a similar motivation. In this context, a typical problem is to compute (possibly approximately) some statistics or an aggregate function over the sequence of data, where the main point is to assume severe restrictions on the space usage. Note that in our setting, we focus on obtaining low time complexity per update and query, rather than optimizing the space complexity. In this respect, our work leans more towards the area of dynamic data structures, in particular dynamic query evaluation [15, 16]. For Boolean properties several papers [17–19] have considered streaming algorithms for testing membership in regular and context-free languages. Another variant of the problem was considered in [20–22], where the regular property is verified on the last *N* letters of the stream, instead of the entire prefix up to the current position.

The closest to our setting is the work [23], which studies the dynamic evaluation problem for monoids over a sliding window, and describes a data structure that can be updated in constant time for a fixed finite monoid. When the monoid is finite, the considered problem is basically the same as monitoring whether the input stream restricted to the sliding window is accepted by a finite automaton. We show in Example 1, that in this case, the problem can be reduced to the dynamic acceptance problem for a special form of timed automaton.

## Preliminaries

*Finite automata* A *finite automaton* is a tuple ${\mathcal {A}}= (\Sigma , Q, I, E, F)$, where $\Sigma$ is a finite alphabet, *Q* is a finite set of states, $E \subseteq Q \times \Sigma \times Q$ is a transition relation, and $I,F \subseteq Q$ are the sets of initial and final states. A run of ${\mathcal {A}}$ on a word $w = a_1 \ldots a_n \in \Sigma ^*$ is a sequence $\rho = q_0 \mathop {\longrightarrow }\limits ^{a_1} q_1\mathop {\longrightarrow }\limits ^{a_2} \ldots \mathop {\longrightarrow }\limits ^{a_n} q_n$ where $(q_{i-1}, a_i, q_i) \in E$ for all $i=1,\dots ,n$. Moreover, $\rho$ is a *successful* run if $q_0 \in I$ and $q_n \in F$. A word *w* is *accepted* by ${\mathcal {A}}$ if there is a successful run of ${\mathcal {A}}$ on *w*.

*Timed automata* Let *X* be a finite set of clocks, usually denoted $\mathtt {x},\mathtt {y},\ldots$. A *clock valuation* is a function $\nu : X \rightarrow {\mathbb {R}}_{\ge 0}$ from clocks to non-negative reals. *Clock conditions* are formulas defined by the grammar: $C_X ~:=~ \mathtt {true}\mid \mathtt {x} < c \mid \mathtt {x} > c \mid \mathtt {x} = c \mid (C_X \wedge C_X) \mid (C_X \vee C_X),$ where $\mathtt {x} \in X$ and $c \in {\mathbb {R}}_{\ge 0}$. By a slight abuse of notation, we also denote by $C_X$ the set of clock conditions over *X*. Given a clock condition $\gamma$ and a valuation $\nu$, we say that $\nu$ *satisfies* $\gamma$ and write $\nu \models \gamma$, if the arithmetic expression obtained from $\gamma$ by substituting each clock $\mathtt {x}$ with its value $\nu (\mathtt {x})$ evaluates to true.

A *timed automaton* is a tuple ${\mathcal {A}}= (\Sigma ,Q,X,I,E,F)$, where $\Sigma$, *Q*, *I*, *F* are defined exactly as for finite automata, *X* is a finite set of clocks, and $E \subseteq Q \times \Sigma \times C_X \times Q \times 2^X$ is a finite transition relation. We say that $c\in {\mathbb {R}}_{\ge 0}$ is a *clock constant* of ${\mathcal {A}}$ if *c* appears in some clock condition of a transition from *E*. A *configuration* of ${\mathcal {A}}$ is a pair $(q, \nu )$, where $q \in Q$ and $\nu$ is a clock valuation. Recall that finite automata process words over a finite alphabet $\Sigma$; likewise, timed automata process timed words over an alphabet of the form $\Sigma \uplus {\mathbb {R}}_{> 0}$, with $\Sigma$ finite.

A *run* of a timed automaton ${\mathcal {A}}$ on a timed word $w = e_1 \ldots e_n \in (\Sigma \cup {\mathbb {R}}_{> 0})^*$ is a sequence $\rho = (q_0,\nu _0) \mathop {\longrightarrow }\limits ^{e_1} (q_1,\nu _1)\mathop {\longrightarrow }\limits ^{e_2} \ldots \mathop {\longrightarrow }\limits ^{e_n} (q_n,\nu _n)$, where each $(q_i, \nu _i)$ is a configuration and

- if $e_i \in {\mathbb {R}}_{> 0}$, then $q_{i+1} = q_i$ and $\nu _{i+1}(\mathtt {x}) = \nu _{i}(\mathtt {x}) + e_i$ for all $\mathtt {x} \in X$;
- if $e_i \in \Sigma$, then there is a transition $(q_i,e_i,\gamma ,q_{i+1},Z) \in E$ such that $\nu _i \models \gamma$ and either $\nu _{i+1}(\mathtt {x})=0$ or $\nu _{i+1}(\mathtt {x})=\nu _{i}(\mathtt {x})$ depending on whether $\mathtt {x}\in Z$ or $\mathtt {x}\in X{\setminus } Z$.

Thus, the set *Z* in a transition $(q_i,e_i,\gamma ,q_{i+1},Z) \in E$ corresponds to the subset of clocks that are reset when firing the transition. Note that the values of the other clocks stay unchanged. An example of a one clock timed automaton was given in the introduction (see Fig. 1).

A run $\rho$ as above is *successful* if $q_0\in I$, $\nu _0(\mathtt {x})=0$ for all $\mathtt {x}\in X$, and $q_n \in F$. A word $w\in (\Sigma \cup {\mathbb {R}}_{> 0})^*$ is *accepted* by ${\mathcal {A}}$ if there is a successful run of ${\mathcal {A}}$ on *w*.

*Size of an automaton* The size of a finite automaton ${\mathcal {A}}= (\Sigma , Q, I, E, F)$ is defined as $|{\mathcal {A}}| = |Q| + |E|$. This is asymptotically equivalent to essentially every possible definition of size of a finite automaton that can be found in the literature. The size of a timed automaton ${\mathcal {A}}= (\Sigma ,Q,X,I,E,F)$ is instead defined as $|{\mathcal {A}}| = |Q| + |X| + \sum _{(p,a,\gamma ,q,Z) \in E} |\gamma |$, where $|\gamma |$ is the number of atomic expressions (*i.e.*, expressions of the form $\mathtt {true}$, $\mathtt {x} < c$, $\mathtt {x} > c$, $\mathtt {x} = c$) appearing in the clock condition $\gamma$. *Note that the size of a timed automaton does not take into account the magnitude of the clock constants.* These constants are specified with the automaton and stored in suitable floating-point memory cells (see the computation model below).

*Computation model* As clock constants and time spans in the input stream are arbitrary real numbers, it is convenient to use the *real RAM model* of computation [24]. This is a standard model with integer memory cells that can store integers and floating-point memory cells that can store real numbers. There are no bounds on the bit length or precision of the stored numbers. However, in practice, our model will only receive inputs with real numbers that are represented symbolically (*e.g.*, rational numbers). Basic arithmetic operations — addition, subtraction, multiplication, and division — can be performed in unit time, but modulo arithmetics and rounding are not included in the model. In fact, we do not use multiplication or division on real numbers either.

## The Dynamic Acceptance Problem

The *dynamic acceptance problem* amounts to designing a data structure that can be initialized for a given timed automaton ${\mathcal {A}}$ with one clock, and afterwards, upon consuming consecutive elements of the data stream, efficiently maintains the information on whether the word read so far is accepted by ${\mathcal {A}}$. Formally, the data structure should support the following operations:

- ${\mathtt {init}}_{}({\mathcal {A}})$: Initialize the data structure for a given automaton ${\mathcal {A}}$. This automaton is fixed for the entire lifespan of the data structure.
- ${\mathtt {accepted}}$: Query whether the prefix of the stream consumed up to the current moment is accepted by ${\mathcal {A}}$.
- $\mathtt {read}_{}{(e)}$: Consume the next element *e* from the input stream, be it a letter from $\Sigma$ or a time span from ${\mathbb {R}}_{> 0}$, and update the data structure accordingly.

The running time of each of these operations needs to be as low as possible. More precisely, we shall say that a data structure *supports dynamic acceptance in time f(s, n)* if the first operation ${\mathtt {init}}_{}({\mathcal {A}})$ takes at most *f*(*s*, 0) time, and every subsequent execution of ${\mathtt {accepted}}$ or $\mathtt {read}_{}{(e)}$ takes at most *f*(*s*, *n*) time, where $s=|{\mathcal {A}}|$ and *n* is the number of stream elements consumed so far. Similarly, a data structure *supports dynamic acceptance in amortized time f(s, n)* if the first operation ${\mathtt {init}}_{}({\mathcal {A}})$ takes at most *f*(*s*, 0) time and, for every *n*, the first *n* operations of the form ${\mathtt {accepted}}$ and $\mathtt {read}_{}{(e)}$ take at most $n\cdot f(s,n)$ time in total. Ultimately, we are interested in designing data structures where the complexity guarantee *f*(*s*, *n*) is independent of *n*, that is, the (amortized) update time is a function of $|{\mathcal {A}}|$ only.

Before presenting the complexity results in detail, we provide an example of application of the dynamic acceptance problem.

### Example 1

We discuss the relationship between our dynamic acceptance problem for timed automata and an aggregation problem for monoids over a sliding window, as considered in [23]. When the monoid is finite, every element of it represents a regular language, and thus the aggregation problem can be seen as an acceptance problem. This means that the aggregation problem for finite monoids over a sliding window is reducible to an automaton acceptance problem in the *sliding window model* (see also [22]). We formalize this problem below.

Let ${\mathcal {A}}= (\Sigma , Q, I, E, F)$ be a finite automaton and *C* a positive integer defining the width of the sliding window. The acceptance problem of ${\mathcal {A}}$ with a sliding window of width *C* consists of processing, from left to right, an arbitrary input $w=a_1 a_2 a_3 \ldots$ over $\Sigma$, while maintaining the answer to the following query: *is the sequence of the last* *C* *consumed letters accepted by* ${\mathcal {A}}$? The goal is to design a data structure that, upon consuming a new letter of a potentially infinite stream, can be updated in a time that only depends on the automaton ${\mathcal {A}}$, and not on size of the window *C*.

**Fig. 2:**
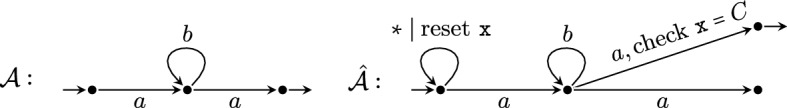
Reducing the sliding window acceptance problem to the dynamic acceptance problem

Next, we explain how the above problem can be reduced to our dynamic acceptance problem. Here, we consider only streams that are discrete, and in fact even slightly more restricted: we assume that every input stream belongs to the language $(\{1\} \cdot \Sigma )^\omega$, namely, that the letters from $\Sigma$ are interleaved by the time unit 1. We map the input word $w = a_1a_2a_3\ldots$ to a corresponding discrete stream ${\widehat{w}}= 1 a_1 1 a_2 1 a_3 \ldots$, and modify the finite automaton ${\mathcal {A}}$ to obtain a corresponding timed automaton ${\widehat{{\mathcal {A}}}}$, as follows. We introduce a new state ${\widehat{q}}$, which will be the only final state of ${\widehat{{\mathcal {A}}}}$, and a clock $\mathtt {x}$. We then replace every transition $(q,a,q')$ of ${\mathcal {A}}$ with the transition $(q,a,\mathtt {true},q',\varnothing )$. Note that these transitions have a vacuous clock condition, hence they are applicable in ${\widehat{{\mathcal {A}}}}$ whenever the original transitions of ${\mathcal {A}}$ are so. In addition, when the former transition $(q,a,q')$ reaches a final state $q'\in F$, we also have a transition $(q,a,\mathtt {x}=C,{\widehat{q}},\varnothing )$ in ${\widehat{{\mathcal {A}}}}$. Finally, we add looping transitions on the initial states that reset the clock, that is, transitions of the form $(q,a,\mathtt {true},q,\{\mathtt {x}\})$, with $q\in I$ and $a \in \Sigma$. Figure 2 shows the timed automaton ${\widehat{{\mathcal {A}}}}$ corresponding to an automaton ${\mathcal {A}}$ recognising $ab^*a$.

From the above construction it is clear that ${\widehat{{\mathcal {A}}}}$ accepts a prefix $1 a_1 \dots 1 a_n$ of ${\widehat{w}}$ if and only if ${\mathcal {A}}$ accepts the *C*-letter factor $a_{n-C+1} \dots a_n$ of *w*. Thus, the acceptance problem for ${\mathcal {A}}$ in the *C*-width sliding window model is reduced to the dynamic acceptance problem for ${\widehat{{\mathcal {A}}}}$ over the stream ${\widehat{w}}$. We will see later (Theorem 1) that there is a data structure that supports dynamic acceptance for ${\widehat{{\mathcal {A}}}}$ with update time ${\mathcal {O}}(2^{3|{\widehat{{\mathcal {A}}}}|})={\mathcal {O}}(2^{6|{\mathcal {A}}|})$. This means that we can process one letter at a time from a word *w*, while answering in time ${\mathcal {O}}(2^{6|{\mathcal {A}}|})$ whether ${\mathcal {A}}$ accepts the sequence of the last *C* consumed letters. Note that the complexity here is independent of the parameter *C*.

### Example 2

Consider a scenario from *complex event processing* (CEP), with a specification language called CEL and defined by the following grammar [25, 26]:where $a \in \Sigma$ and $t \in {\mathbb {N}}$. A word $w=a_1 a_2 \ldots a_n \in \Sigma ^*$ *matches* an expression $\varphi$ from the above grammar, denoted $w \vDash \varphi$, if one of the following cases holds:

- $$\varphi = a_n$$,
, $$w = w_1 \cdot w_2$$, $$w_1 \vDash \varphi _1$$, and $$w_2 \vDash \varphi _2$$,
- $$\varphi = (\varphi ' {{\,\mathrm{\textsc {Within}}\,}}t)$$ and $$a_m\ldots a_n \vDash \varphi '$$, where $$m = \max \{1,n-t\}$$.

**Fig. 3:**
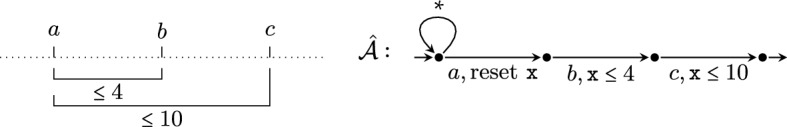
Translation of a CEL expression into an equivalent single-clock timed automaton

Given a word $w=a_1 a_2 \dots$ and an expression $\varphi$, we would like to read *w* sequentially, as in a stream, and decide, at each position $n=1,2,\dots$, whether the prefix $w_n = a_1 \dots a_n$ matches a fixed expression $\varphi$. One can reduce this latter problem to the dynamic acceptance problem for timed automata, by using a discrete timed word ${\widehat{w}}= 1 a_1 1 a_2 1 \dots$ as before and by translating the expression $\varphi$ into an appropriate timed automaton. We omit the straightforward details of the translation of a CEL expression to an equivalent timed automaton, and we only remark that every occurrence of the ${{\,\mathrm{\textsc {Within}}\,}}$ operator in an expression corresponds to a condition on a specific clock in the equivalent timed automaton. This means that, in general, the translation may require a timed automaton with multiple clocks. However, there are simple cases where, even in the presence of nested ${{\,\mathrm{\textsc {Within}}\,}}$ operators, one can construct an equivalent timed automaton with a single clock. More precisely, this is possible when, for every occurrence of a ${{\,\mathrm{\textsc {Within}}\,}}$ operator, say $(\varphi {{\,\mathrm{\textsc {Within}}\,}}t)$, and for every sub-expression of $\varphi$ of the form , $\varphi _2$ does not contain a ${{\,\mathrm{\textsc {Within}}\,}}$ operator. This is the case, for instance, of the expression . This expression describes a sequence containing three (possibly not contiguous) events *a*, *b*, *c*, with *a* and *b* at distance at most 4 and *a* and *c* at distance at most 10. Figure 3 shows a single-clock timed automaton that is equivalent to $\varphi$, in the sense that it accepts a timed word of the form $1a_11a_21\ldots 1a_n$ if and only if $a_1a_2 \ldots a_n \vDash \varphi$. In this case one can validate any input stream against a fixed expression $\varphi$ in time that is constant per input letter, by simply reducing to our dynamic acceptance problem for single-clock timed automata and discrete timed words.

*Results* We say that a stream *w* is *discrete* if its elements range over $\Sigma \uplus \{1\}$, that is, if all time spans in the stream coincide with the time unit 1. Our main result is the following:

### Theorem 1

Consider the dynamic acceptance problem for timed automata with one clock. There is a data structure that

- supports dynamic acceptance in time ${\mathcal {O}}(2^{3|{\mathcal {A}}|})$ on discrete streams, and
- supports dynamic acceptance in amortized time ${\mathcal {O}}(2^{3|{\mathcal {A}}|})$ on arbitrary streams,

where ${\mathcal {A}}$ is the automaton provided upon initialization.

We stress that the complexity in Theorem 1 depends only on the size of ${\mathcal {A}}$. In particular, it does not depend on the bit-length of clock constants (*e.g.*, constant 24 in Fig. 1). Note that thanks to the assumption of the real RAM model, the question of the complexity of arithmetic operations on reals is separated from the running time analysis in the Proof of Theorem 1. This feature reflects the real-life scenarios, where the automaton is small, while real numbers involved can be efficiently manipulated by the processor despite having large bit-length The Proof of Theorem 1 is presented in Sect. 4.

We do not know whether this theorem can be generalized to timed automata with more than one clock while preserving independence of the time complexity of updates from the length of the consumed stream prefix. However, we establish a negative result for a slightly more powerful model of timed automata, called timed automata with additive constraints (see *e.g.*, [5]). Formally, a *timed automaton with additive constraints* is defined exactly as a timed automaton — that is, as a tuple ${\mathcal {A}}= (\Sigma ,Q,X,I,E,F)$ consisting of an input alphabet, a set of states, a set of clocks, etc. — but clock conditions are now allowed to satisfy an extended grammar obtained by adding new rules of the form $(\sum _{\mathtt {x}\in Z} \mathtt {x}) \sim c$, where $Z\subseteq X$ and $\sim \; \in \{<, >, =\}$. For instance, one can write $\mathtt {x}+\mathtt {y}\le c$, where *c* is a clock constant. To give some background, let us briefly discuss in more detail the power of this extension. Allowing additive constraints is a non-trivial extension of timed automata and in particular it makes the emptiness problem undecidable [5,  Theorem 2]. However, undecidability holds when at least four clocks are available. Moreover, it is shown that for timed automata with additive constraints with two clocks the emptiness problem is decidable; and the proof is a straightforward modification of the standard region construction [5,  Proposition 1].

Our negative result relies on the 3SUM Conjecture, stated just below. Recall that in the 3SUM problem we are given a set *S* of positive real numbers and the question is to determine whether there exist $a,b,c\in S$ satisfying $a+b=c$. It is easy to solve the problem in time ${\mathcal {O}}(n^2)$, where $n=|S|$; the 3SUM Conjecture asserts that this cannot be significantly improved:

[3SUM Conjecture] In the real RAM model, the 3SUM problem cannot be solved in strongly sub-quadratic time, that is, in time ${\mathcal {O}}(n^{2-\delta })$ for any $\delta >0$, where *n* is the number of values forming the

### Theorem 2

If the 3SUM Conjecture holds, then there is a two-clock timed automaton ${\mathcal {A}}$ with additive constraints such that there is no data structure that, when initialized on ${\mathcal {A}}$, supports dynamic acceptance in time ${\mathcal {O}}(n^{1-\delta })$ for any $\delta >0$, where *n* is the length of the consumed stream prefix.

A detailed discussion on the 3SUM Conjecture and the Proof of Theorem 2 are postponed to Sect. 5.

## Data Structure: Proof of Theorem 1

*Notation* Let us fix, once and for all, the timed automaton ${\mathcal {A}}= (\Sigma ,Q,X,I,E,F)$ with a single clock $\mathtt {x}$ that is provided upon initialization. By adding a non-accepting sink state, if necessary, we may assume that for every $q\in Q$ and $a\in \Sigma$, some transition over letter *a* can be always applied at *q* at any time (with a trivial clock condition). Note that this means that the number of runs never decreases over time.

As ${\mathcal {A}}$ uses only one clock $\mathtt {x}$, every configuration of ${\mathcal {A}}$ can be written simply as a pair (*q*, *t*), where $q\in Q$ is the state and $t\in {\mathbb {R}}_{\ge 0}$ is the value of the clock $\mathtt {x}$. Let $0=C_0<C_1<\ldots <C_m$ be the clock constants used in ${\mathcal {A}}$, where we assume without loss of generality that $C_0=0$. For simplicity we also let $C_{m+1}=\infty$. Note that $m\le |{\mathcal {A}}|$.

Consider now an arbitrary stream $w \in (\Sigma \cup {\mathbb {R}}_{> 0})^\omega$. For every $n\in {\mathbb {N}}$, let $w_n = w[1\ldots n]$ be the *n*-element prefix of *w*. Recall that $w_n$ can be thought of as the stream prefix that is disclosed after *n* operations $\mathtt {read}_{}{(e)}$. We say that a configuration (*q*, *t*) is *active* at step *n* if there is a run of ${\mathcal {A}}$ on $w_n$ that starts in a configuration $(q_0,0)$ for some $q_0\in I$ and ends in (*q*, *t*). We let $K_n$ be the set of all configurations (*q*, *t*) that are active at step *n*.

*Partitioning the problem* It is clear that the dynamic acceptance problem essentially boils down to designing an efficient data structure that maintains $K_n$ upon reading subsequent elements from the stream. This data structure should offer a query on whether $K_n$ contains an accepting configuration. The main observation is that any two clock values *t* and $t'$ that are in the same relative order with respect to the clock constants $C_1,\ldots ,C_m$ (*i.e.*, for every $i=1,\dots ,m$, $t < C_i$ iff $t' < C_i$, and similarly $t \le C_i$ iff $t' \le C_i$) satisfy exactly the same clock conditions in *E*. Precisely, let us consider the partition of ${\mathbb {R}}_{\ge 0}$ into intervals $J_0$, $J_1$, ...$J_{2m+1}$, where $J_{2i} = [C_i,C_i]$, $J_{2i+1} = (C_{i}, C_{i+1})$, for all $i \in \{0,\ldots ,m\}$. The following assertion holds: for any two configurations (*q*, *t*), $(q,t')$, with $t,t'\in J_i$ for some $0\le i\le 2m+1$, exactly the same transitions are available in (*q*, *t*) as in $(q,t')$.

For $n\in {\mathbb {N}}$ and $i\in \{0,\ldots ,2m+1\}$, let

$$\begin{aligned} K_n[i] = \{\,(q,t)\in K_n\ :\ t\in J_i\,\}. \end{aligned}$$

The idea is to maintain each set $K_n[i]$ in a separate data structure. Each of these data structures follows the same design, which we call the *inner data structure*.

*Inner data structure: an overview* Every inner data structure is constructed for an interval $J\in \{J_0,\ldots ,J_{2m+1}\}$. We will denote it by ${\mathbb {D}}[J]$, or simply by ${\mathbb {D}}[i]$ when $J=J_i$. Each structure ${\mathbb {D}}[J]$ stores a set of configurations $L$ satisfying the following invariant: all clock values of configurations in $L$ belong to *J*. In the final design we will maintain the invariant that the set $L$ stored by ${\mathbb {D}}[i]$ at step *n* is equal to $K_n[i]$, but for the design of ${\mathbb {D}}[J]$ it is easier to treat $L$ as an arbitrary set of configurations with clock values in *J*.

The inner data structure should support the following methods:

- Method ${\mathtt {init}}(J)$ stores interval *J* and initializes ${\mathbb {D}}[J]$ by setting $L=\varnothing$.
- Method ${\mathtt {accepted}}()$ returns true or false, depending on whether or not $L$ contains an accepting configuration, that is, a configuration (*q*, *t*) such that $q\in F$.
- Method ${\mathtt {insert}}(q,t)$ adds a configuration (*q*, *t*) to $L$. This method will be always applied with a promise that $t\in J$ and $t\le t'$ for all configurations $(q',t')$ already present in $L$.
- Method ${\mathtt {updateTime}}(r)$, where $r\in {\mathbb {R}}_{>0}$, increments the clock values of all configurations in $L$ by *r*. All configurations whose clock values ceased to belong to *J* are removed from $L$, and they are returned by the method on output. This output is organised as a doubly linked list of configurations, sorted by non-decreasing clock values.
- Method ${\mathtt {updateLetter}}(a)$ updates $L$ by applying to all configurations in $L$ all possible transitions over the given letter $a\in \Sigma$. Precisely, the updated set comprises all configurations (*q*, *t*) that can be obtained from configurations belonging to $L$ before the update using transitions over *a* that do not reset the clock. The configurations (*q*, 0) which can be obtained from $L$ using transitions over *a* that do reset the clock are not included in the updated set, but are instead returned by the method as a doubly linked list.

In Sect. 4.2 we will provide an efficient implementation of the inner data structure, which is encapsulated in the following lemma.

### Lemma 3

For each $J\in \{J_0,J_1,\ldots ,J_{2m+1}\}$, the inner data structure ${\mathbb {D}}[J]$ can be implemented so that methods ${\mathtt {init}}()$, ${\mathtt {accepted}}()$, ${\mathtt {insert}}(\cdot ,\cdot )$, and ${\mathtt {updateLetter}}(\cdot )$ run in time ${\mathcal {O}}(2^{|{\mathcal {A}}|})$, while method ${\mathtt {updateTime}}(\cdot )$ runs in time ${\mathcal {O}}(2^{|{\mathcal {A}}|})\cdot \ell$, where $\ell$ is the size of its output.

We postpone the Proof of Lemma 3 and we show now how to use it to prove Theorem 1. That is, we design an *outer data structure* that monitors the acceptance of ${\mathcal {A}}$.

### Outer Data Structure

The outer data structure consists of a list ${\mathbb {D}}[0],\ldots ,{\mathbb {D}}[2m+1]$, where each ${\mathbb {D}}[i]$ is a copy of the inner data structure constructed for the interval $J_i$. We will keep the following invariant:

1. After step *n*, for each $i\in \{0,1,\ldots ,2m+1\}$ the data structure ${\mathbb {D}}[i]$ stores $K_n[i]$.

We first explain how the outer data structure implements the promised operations: initialization, queries about the acceptance, and updates upon reading the next element of the stream *w*. Then we discuss the amortized complexity of the updates.

*Initialization* Given ${\mathcal {A}}$, we store ${\mathcal {A}}$ in the data structure and we read the clock constants $0=C_0<C_1<\ldots <C_m$ from ${\mathcal {A}}$. Then we initialize $2m+1$ copies ${\mathbb {D}}[0],\ldots ,{\mathbb {D}}[2m+1]$ of the inner data structure by calling method ${\mathtt {init}}(J)$ for each interval *J* among $J_0,J_1,\dots ,J_{2m+1}$. Finally, for each initial state *q*, we apply method ${\mathtt {insert}}(q,0)$ on ${\mathbb {D}}[0]$. As $K_0=\{(q,0)\ :\ q\in I\}$, after this we have that Invariant (I1) holds for $n=0$.

*Query* We query all the data structures ${\mathbb {D}}[0],\ldots ,{\mathbb {D}}[2m+1]$ for the existence of accepting configurations using the ${\mathtt {accepted}}()$ method, and return the disjunction of the answers. The correctness follows directly from Invariant (I1).

*Update by a time span* Suppose the next element from the stream is a time span $r\in {\mathbb {R}}_{> 0}$. We update the outer data structure as follows. First, we apply method ${\mathtt {updateTime}}(r)$ to each data structure ${\mathbb {D}}[i]$. This operation increments the clock values of all configurations stored in ${\mathbb {D}}[i]$ by *r*, but may output a set of configurations whose clock values ceased to fit in the interval $J_i$. Recall that this set is organised as a doubly linked list of configurations, sorted by non-decreasing clock values; call this list $S_i$. Now, we need to insert each configuration (*q*, *t*) that appears on those lists into the appropriate data structure ${\mathbb {D}}[j]$, where *j* is such that $t\in J_j$. However, we have to be careful about the order of insertions: we process the lists $S_{2m+1},S_{2m},\ldots ,S_0$ in this precise order, and each list $S_i$ is processed from the end, that is, following the non-increasing order of clock values. When processing a configuration (*q*, *t*) from the list $S_i$, we find the index $j>i$ such that $t\in J_j$ and apply the method ${\mathtt {insert}}(q,t)$ on the structure ${\mathbb {D}}[j]$. In this way the condition required by the ${\mathtt {insert}}$ method — that $t\le t'$ for every configuration $(q',t')$ currently stored in ${\mathbb {D}}[j]$ — is satisfied. It is also easy to see that Invariant (I1) is preserved after the update.

*Update by a letter* Suppose the next symbol read from the stream is a letter $a\in \Sigma$. We update the outer data structure as follows. First, we apply method ${\mathtt {updateLetter}}(a)$ to each data structure ${\mathbb {D}}[i]$. This operation applies all possible transitions on letter *a* to all configurations stored in ${\mathbb {D}}[i]$, and outputs a list of configurations $R_i$ where the clock got reset. All these configurations have clock value 0, hence the length of $R_i$ is at most $|Q|$. It now suffices to insert all the configurations (*q*, 0) appearing on all the lists $R_i$ to ${\mathbb {D}}[0]$ using method ${\mathtt {insert}}(q,0)$. We may do this in any order, as the condition required by the ${\mathtt {insert}}$ method is trivially satisfied. Again, Invariant (I1) is clearly preserved after the update.

This concludes the implementation of the outer data structure. While the correctness is clear from the description, we are left with arguing that the time complexity is as promised.

From Lemma 3 it readily follows that each of the following operations takes time ${\mathcal {O}}(2^{|{\mathcal {A}}|})$: initialization, a query about the acceptance, and an update by a letter. As for an update by a time span $r\in {\mathbb {R}}_{> 0}$, by Lemma 3 the complexity of such an update is ${\mathcal {O}}(2^{|{\mathcal {A}}|})\cdot \sum _{i=0}^{2m+1} |S_i|$, where $S_0,\ldots ,S_{2m+1}$ are the sets returned by the applications of method ${\mathtt {updateTime}}(r)$ to data structures ${\mathbb {D}}[0],\ldots ,{\mathbb {D}}[2m+1]$, respectively. We need to argue that the amortized time complexity of all these updates is bounded by ${\mathcal {O}}(2^{|{\mathcal {A}}|})$.

Consider the following definition: a clock value $t\in {\mathbb {R}}_{\ge 0}$ is *active* at step *n* if $K_n$ contains a configuration with clock value *t*. Observe that upon an update by a time span $r\in {\mathbb {R}}_{> 0}$, the set of active clock values simply gets shifted by *r*, while upon an update by a letter $a\in \Sigma$ it stays the same, except that clock value 0 may also become active. Since at step 0 the only active clock value is 0, we conclude that for every $n\in {\mathbb {N}}$, at most $n+1$ active clock values may have appeared until step *n*. Note that there may be at most $|Q|$ different active configurations with the same active clock value, hence the complexity of each update by a time span is bounded by ${\mathcal {O}}(2^{|{\mathcal {A}}|})\cdot |Q|$ times the number of active clock values that change membership from an interval to another one, where we imagine that each active clock value is shifted by the time span. Since every active clock value can change membership in an interval at most $2m+1$ times, and since the total number of active values that appear until step *n* is at most $n+1$, we derive that the total time spent on updates by time spans throughout the first *n* steps is bounded by ${\mathcal {O}}(2^{|{\mathcal {A}}|})\cdot |Q|\cdot (2m+1)\cdot (n+1)$. Hence, by recalling that $|Q|,m \le |{\mathcal {A}}|$, we conclude that the amortized time complexity is ${\mathcal {O}}(2^{3|{\mathcal {A}}|})$.

Finally, note that in the case of discrete streams each set $S_i$ consists of configurations with the same clock value, hence $|S_i|\le |Q|\le |{\mathcal {A}}|$ for all $i\in \{0,\ldots ,2m+1\}$. So in this case, the complexity of an update by a time span is bounded by ${\mathcal {O}}(2^{3|{\mathcal {A}}|})$, without any amortization.

This finishes the Proof of Theorem 1, assuming Lemma 3. We prove the latter next.

### Inner Data Structure

We now describe the inner data structure ${\mathbb {D}}[J]$ and prove Lemma 3. Let us fix an interval $J\in \{J_0,\ldots ,J_{2m+1}\}$. We denote by $L$ the set of configurations currently stored by the inner data structure ${\mathbb {D}}[J]$. It is convenient to represent $L$ by a function $\lambda :{\mathbb {R}}_{\ge 0}\rightarrow 2^Q$ defined by

$$\begin{aligned} \lambda (t)=\{\,q\in Q\ :\ (q,t)\in L\}. \end{aligned}$$

We let ${\widehat{L}}$ be the set of all clock values that are *active* in $L$, that is, ${\widehat{L}}$ comprises all $t\in {\mathbb {R}}_{\ge 0}$ such that $\lambda (t)\ne \varnothing$. Recall that we assume that ${\widehat{L}}\subseteq J$.

Before we dive into the details, let us discuss the intuition. The basic idea is to store all the configurations in $L$ in a queue, implemented as a doubly-linked list ordered by non-decreasing clock values. To handle clock values efficiently, we do not store them directly. Instead, we maintain a global clock that measures the total time since the initialization of the data structure, and each configuration bears a timestamp that is the value of this global clock at the moment of the last reset. Thus, updating by a time span boils down to increasing the value of the global clock and popping any configurations at the back of the queue whose clock values ceased to fit into the interval *J*.

Updating by a letter is more problematic, as we need to apply the transition relation of the automaton ${\mathcal {A}}$ to all the configurations of $L$ simultaneously. In the data structure we store a partition of the active clock values ${\widehat{L}}$ according to their images under $\lambda (\cdot )$, so that for each block of this partition (whose number is at most $2^{|Q|}$), we can simultaneously update all corresponding configurations in constant time. There is a caveat here: it is possible that for some $t,t'\in {\widehat{L}}$ we have $\lambda (t)\ne \lambda (t')$ before the update, but $\lambda (t)=\lambda (t')$ after the update. That is, the blocks of the partition may require merging upon updates. We resolve this issue by representing the partition in a *forest*, similarly as the union-find data structure would do. The key point is that the height of this forest can be kept bounded by $2^{|Q|}$.

*Description of the structure* In short, the data structure ${\mathbb {D}}[J]$ consists of three elements:

- The *clock*, denoted ${\mathtt {y}}$, is a real that represents the total time elapsed since initialization.
- The *list*, denoted ${\mathtt {l}}$, stores the set of active clock values ${\widehat{L}}$.
- The *forest*, denoted ${\mathtt {f}}$, is built on top of the elements of ${\mathtt {l}}$ and describes the function $\lambda$.

**Fig. 4:**
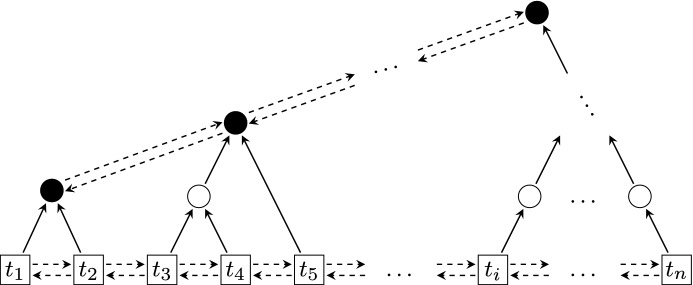
The inner data structure. List elements are depicted as squares while the forest nodes are depicted as circles. The black circles are the roots

We describe the list and the forest in more details (the reader can refer to Fig. 4).

*The list* The list ${\mathtt {l}}$ encodes the clock values present in ${\widehat{L}}$, sorted in the increasing order and organised into a doubly linked list. Each node $\alpha$ on ${\mathtt {l}}$ is a record consisting of:

- ${\mathtt {next}}(\alpha )$: a pointer to the next node on the list;
- ${\mathtt {prev}}(\alpha )$: a pointer to the previous node on the list; and
- ${\mathtt {timestamp}}(\alpha )\in {\mathbb {R}}$: the *timestamp* of the node.

As usual, the data structure stores ${\mathtt {l}}$ by maintaining pointers to the first and last nodes.

The clock value represented by a node $\alpha$ on ${\mathtt {l}}$ is equal to ${\mathtt {clock}}(\alpha ) = {\mathtt {y}}- {\mathtt {timestamp}}(\alpha )$; this will always be a non-negative real. Thus, the timestamp is essentially the total elapsed time recorded at the moment of the last reset of the clock. Note that this implementation allows for a simultaneous increment of ${\mathtt {clock}}(\alpha )$ for all nodes $\alpha$ on ${\mathtt {l}}$ in constant time: it suffices to simply increment ${\mathtt {y}}$.

*The forest* Forest ${\mathtt {f}}$ represents the mapping from elements $t\in {\widehat{L}}$, encoded in ${\mathtt {l}}$, to respective sets of control states $\lambda (t)$. It is a rooted forest where nodes may have arbitrarily many children, and these children are unordered. Every node $\gamma$ of ${\mathtt {f}}$ is a record containing:

- ${\mathtt {parent}}(\gamma )$: a pointer to the parent of $\gamma$; and
- ${\mathtt {\#children}}(\gamma )$: an integer equal to the number of children of $\gamma$.

The leaves of the forest will always coincide with the nodes on the list ${\mathtt {l}}$. In particular, we augment the records stored for the nodes on ${\mathtt {l}}$ by adding the ${\mathtt {parent}}(\cdot )$ pointer, and treat them as nodes of the forest ${\mathtt {f}}$ at the same time. The counter ${\mathtt {\#children}}(\cdot )$ would always be equal to 0 for those nodes, so we may omit it.

The *roots* of the forest are the nodes $\beta$ with no parent, *i.e.*, ${\mathtt {parent}}(\beta )=\bot$. We will maintain the invariant that no root is a leaf in ${\mathtt {f}}$, that is, every root has at least one child. In the data structure we store a doubly linked list containing all the roots of ${\mathtt {f}}$. This list will be denoted ${\mathtt {r}}$, and again it is stored by pointers to its first and last element. Thus, the records of the roots of ${\mathtt {f}}$ are augmented by ${\mathtt {next}}(\cdot )$ and ${\mathtt {prev}}(\cdot )$ pointers describing the structure of ${\mathtt {r}}$, with the usual meaning. In addition to this, every root $\beta$ of ${\mathtt {f}}$ carries two additional values:

- ${\mathtt {states}}(\beta )\subseteq Q$: a non-empty subset of control states for which $\beta$ is responsible; and
- ${\mathtt {rank}}(\beta )$: an integer from the set $\{1,2,3,\ldots ,2^{|Q|}\}$.

We will maintain two invariants about these values. First, the sets ${\mathtt {states}}(\beta )$ must be different for distinct roots $\beta$ of ${\mathtt {f}}$, and the same holds for the ranks ${\mathtt {rank}}(\beta )$. Note that this implies that ${\mathtt {f}}$ has at most $2^{|Q|}-1$ roots. Second, for every root $\beta$, the *tree rooted at* $\beta$ — which is the tree containing $\beta$ and all its descendants in ${\mathtt {f}}$ — has depth at most ${\mathtt {rank}}(\beta )+1$ where the *depth* of a forest is the maximum number of edges on a path from a leaf to a root. Note that this implies that the depth of the forest ${\mathtt {f}}$ is bounded by $2^{|Q|} + 1$.

Function $\lambda$ is then represented as follows. For every node $\alpha$ on ${\mathtt {l}}$, let ${\mathtt {root}}(\alpha )$ be the root of the tree of ${\mathtt {f}}$ that contains $\alpha$. Then denoting $t={\mathtt {clock}}(\alpha )$, we have $\lambda (t)={\mathtt {states}}({\mathtt {root}}(\alpha ))$. Note that the invariant stated above implies that from every leaf $\alpha$ of ${\mathtt {f}}$, ${\mathtt {root}}(\alpha )$ can be computed from $\alpha$ by following the ${\mathtt {parent}}(\cdot )$ pointer at most $2^{|Q|} \le 2^{|{\mathcal {A}}|}$ times. Hence, given $t\in {\widehat{L}}$ and a node $\alpha$ on ${\mathtt {l}}$ satisfying $t={\mathtt {clock}}(\alpha )$, we can compute $\lambda (t)$ in time ${\mathcal {O}}(2^{|{\mathcal {A}}|})$.

*Invariants* For convenience, we gather here all the invariants maintained by the inner data structure which we mentioned before:

I2. For each node $\alpha$ on ${\mathtt {l}}$, the value ${\mathtt {clock}}(\alpha ) = \mathtt {y} - {\mathtt {timestamp}}(\alpha )$ belongs to *J*.
I3. The nodes on ${\mathtt {l}}$ are sorted by increasing clock values, or equally by decreasing timestamps. That is, ${\mathtt {timestamp}}(\alpha )>{\mathtt {timestamp}}({\mathtt {next}}(\alpha ))$ for every non-last node $\alpha$ on ${\mathtt {l}}$.
I4. Every root of ${\mathtt {f}}$ has at least one child, and the leaves of ${\mathtt {f}}$ are exactly all the nodes on ${\mathtt {l}}$.
I5. The roots of ${\mathtt {f}}$ carry pairwise different, non-empty sets of control states, and they have pairwise different ranks. Moreover, all the ranks belong to the set $\{1,2,\ldots ,2^{|Q|}\}$.
I6. For each root $\beta$ of ${\mathtt {f}}$, the depth of the tree rooted at $\beta$ is at most ${\mathtt {rank}}(\beta )+1$.

*Implementation* Now we show how to implement the methods ${\mathtt {init}}(J)$, ${\mathtt {accepted}}()$, ${\mathtt {insert}}(q,t)$, ${\mathtt {updateTime}}(r)$, and ${\mathtt {updateLetter}}(a)$ in the data structure. Recall that all these methods should work in time ${\mathcal {O}}(2^{|{\mathcal {A}}|})$, with the exception of ${\mathtt {updateTime}}(r)$ which is allowed to work in time ${\mathcal {O}}(2^{|{\mathcal {A}}|}) \cdot \ell$, where $\ell$ is the size of its output. The description of each method is supplied by a running time analysis and an argumentation of the correctness, which includes a discussion on why the invariants stated above are maintained.

*Removing nodes* Before we proceed to the description of the required methods, we briefly discuss an auxiliary procedure of removing a node from the list ${\mathtt {l}}$ and from the forest ${\mathtt {f}}$, as this procedure will be used several times. Suppose we are given a node $\alpha$ on the list ${\mathtt {l}}$ and we would like to remove it, which corresponds to removing from $L$ all configurations (*q*, *t*) where $t={\mathtt {clock}}(\alpha )$ and $q\in \lambda (t)$. We can remove $\alpha$ from ${\mathtt {l}}$ in the usual way. Then we remove $\alpha$ from ${\mathtt {f}}$ as follows. First, we decrement the counter of children in the parent of $\alpha$. If this counter stays positive then there is nothing more to do. Otherwise, we need to remove the parent of $\alpha$ as well, and accordingly decrement the counter of children in the grandparent of $\alpha$. This can again trigger removal of the grandparent and so on. If eventually we need to remove a root of ${\mathtt {f}}$, we also remove it from the list ${\mathtt {r}}$ in the usual way. Since, by Invariants (I5) and (I6), the depth of ${\mathtt {f}}$ is bounded by $2^{|Q|}+1 = {\mathcal {O}}(2^{|{\mathcal {A}}|})$, the whole procedure can be performed in time ${\mathcal {O}}(2^{|{\mathcal {A}}|})$. It is clear that all the invariants are maintained.

*Initialization* The ${\mathtt {init}}(J)$ method stores the interval *J*, that defines the range of clock values that could be represented in the data structure. It also sets ${\mathtt {y}}=0$ and initializes ${\mathtt {l}}$ and ${\mathtt {r}}$ as empty lists. The correctness and the running time are clear.

*Acceptance query* The ${\mathtt {accepted}}()$ method is implemented as follows. We iterate through the list ${\mathtt {r}}$ to check whether there exists a root $\beta$ of ${\mathtt {f}}$ such that ${\mathtt {states}}({\mathtt {f}})$ contains any accepting state, say *q*. If this is the case, then by Invariant (I4) there is a node $\alpha$ on ${\mathtt {l}}$ satisfying ${\mathtt {root}}(\alpha )=\beta$, hence (*q*, *t*) is an accepting configuration that belongs to $L$, where $t={\mathtt {clock}}(\alpha )$. So we may return a positive answer from the query. Otherwise, all configurations in $L$ have non-accepting states, and we may return a negative answer. Note that since by Invariant (I5) the list ${\mathtt {r}}$ has length at most $2^{|Q|}-1$, the above procedure works in time ${\mathcal {O}}(2^{|{\mathcal {A}}|})$.

*Insertion* We now implement the method ${\mathtt {insert}}(q,t)$, where (*q*, *t*) is a configuration. Recall that when this method is executed, we have a promise that $t\in J$ and $t\le t'$ for all configurations $(q',t')$ that are currently present in ${\mathbb {D}}[J]$.

Let $\alpha$ be the first node on the list ${\mathtt {l}}$. Let $t'={\mathtt {clock}}(\alpha )$. By the promise, we have $t\le t'$. We consider cases: either $t<t'$ or $t=t'$. The former case also captures the situation when ${\mathtt {l}}$ is empty. When $t<t'$ or ${\mathtt {l}}$ is empty, the new configuration (*q*, *t*) gives rise to a new active clock value *t*. Therefore, we create a new list node $\alpha _0$ and insert it at the front of the list ${\mathtt {l}}$. We set the timestamp as ${\mathtt {timestamp}}(\alpha _0)={\mathtt {y}}-t$, so that the node correctly represents the clock value *t*. It is clear that Invariants (I2) and (I3) are thus satisfied.

Next, we need to insert the new node $\alpha _0$ to the forest ${\mathtt {f}}$. We iterate through the list ${\mathtt {r}}$ in search for a root $\beta$ that satisfies ${\mathtt {states}}(\beta )=\{q\}$. In case there is one, we simply set ${\mathtt {parent}}(\alpha _0)=\beta$ and increment ${\mathtt {\#children}}(\beta )$. Otherwise, we construct a new root $\beta _0$ with ${\mathtt {states}}(\beta _0)=\{q\}$ and ${\mathtt {\#children}}(\beta _0)=1$, insert it at the front of the list ${\mathtt {r}}$, and set ${\mathtt {parent}}(\alpha _0)=\beta _0$. To determine the rank of $\beta _0$, we find the smallest integer $k\in \{1,\ldots ,2^{|Q|}\}$ that is *not* used as the rank of any other root of ${\mathtt {f}}$. Observe that, by Invariant (I5), the forest ${\mathtt {f}}$ has at most $2^{|Q|}-1$ roots, so there is always such a number *k*, and it can be found in time ${\mathcal {O}}(2^{|{\mathcal {A}}|})$ by inspecting the list ${\mathtt {r}}$. We then set ${\mathtt {rank}}(\beta _0)=k$. It is clear that this operation can be performed again in time ${\mathcal {O}}(2^{|{\mathcal {A}}|})$, and that Invariants (I4), (I5), and (I6) are maintained. For the last one, observe that the new leaf $\alpha _0$ is attached directly under a root of ${\mathtt {f}}$, so no tree in ${\mathtt {f}}$ existing before the insertion could have increased its depth.

We are left with the case when $t=t'$. We first compute the set *X* equal to $\lambda (t)$ before the insertion: it suffices to find ${\mathtt {root}}(\alpha )$ in time ${\mathcal {O}}(2^{|{\mathcal {A}}|})$ and read $X={\mathtt {states}}({\mathtt {root}}(\alpha ))$. If $q\in X$ then the configuration (*q*, *t*) is already present in $L$, so there is nothing to do. Otherwise, we need to update the data structure so that $\lambda (t)$ is equal to $X\cup \{q\}$ instead of *X*. Consequently, we remove the node $\alpha$ from ${\mathtt {l}}$ and from ${\mathtt {f}}$, using the operation described earlier, and we insert a new node $\alpha '$ at the front of ${\mathtt {l}}$, with the same timestamp equal to that of $\alpha$. Thus, ${\mathtt {clock}}(\alpha ')=t$. We next insert the new node $\alpha '$ to the forest ${\mathtt {f}}$ using the same procedure as described in the previous paragraph, but applied to the state set $X\cup \{q\}$ instead of $\{q\}$. Again, it is clear that these operations can be performed in time ${\mathcal {O}}(2^{|{\mathcal {A}}|})$, and the same argumentation shows that all the invariants are maintained.

*Update by a time span* Next, we implement the method ${\mathtt {updateTime}}(r)$, for $r\in {\mathbb {R}}_{> 0}$. First, we increment ${\mathtt {y}}$ by *r*. Thus, for every node $\alpha$ in the list ${\mathtt {l}}$, the value ${\mathtt {clock}}(\alpha )$ is incremented by *r*. However, the Invariant (I2) may have ceased to hold, as some active clock values could have been shifted outside of the interval *J*. The configurations with these clock values should be removed from the data structure and their list should be the output of the method.

We extract these configurations as follows. Construct an initially empty list of configuration ${\mathtt {lret}}$, on which we shall build the output. Iterate through the list ${\mathtt {l}}$, starting from its back. For each consecutive node $\alpha$, compute $t={\mathtt {clock}}(\alpha )$. If $t\in J$, then break the iteration and return ${\mathtt {lret}}$, as there are no more configurations to remove. Otherwise, find ${\mathtt {root}}(\alpha )$ in time ${\mathcal {O}}(2^{|{\mathcal {A}}|})$, read $\lambda (t)={\mathtt {states}}({\mathtt {root}}(\alpha ))$, and add at the front of ${\mathtt {lret}}$ all configurations (*q*, *t*) for $q\in \lambda (t)$, in any order. Then remove $\alpha$ from the list ${\mathtt {l}}$ and from the forest ${\mathtt {f}}$, and proceed to the previous node in ${\mathtt {l}}$ (if there is none, finish the iteration).

By Invariant (I3), it is clear that in this way we remove from ${\mathbb {D}}[J]$ exactly all the configurations whose clock values got shifted outside of *J*, hence Invariants (I2) and (I3) are maintained. As the forest structure was influenced only by removals, Invariants (I4), (I5), and (I6) are maintained as well. Note that the output list ${\mathtt {lret}}$ is ordered by non-decreasing clock values, as required. As for the time complexity, the procedure presented above takes time ${\mathcal {O}}(2^{|{\mathcal {A}}|}) \cdot \ell '$, where $\ell '$ is the number of nodes that we remove from ${\mathtt {l}}$. As for every node $\alpha$ the set ${\mathtt {states}}({\mathtt {root}}(\alpha ))$ is non-empty and of size at most $|Q|$, with every removed node we add to ${\mathtt {lret}}$ between 1 and $|Q|$ new configurations. Hence, we can also bound the complexity by ${\mathcal {O}}(2^{|{\mathcal {A}}|})\cdot \ell$, where $\ell$ is the number of configurations that appear in the output list ${\mathtt {lret}}$.

*Update by a letter* We proceed to the method ${\mathtt {updateLetter}}(a)$, where $a\in \Sigma$. As argued before, every clock condition appearing in ${\mathcal {A}}$ is either true for all clock values in *J*, or false for all clock values in *J*. For every subset of states $S\subseteq Q$, let $\Phi (S)$ be the set of all states *q* such that there is a transition $(p,a,q,\gamma ,\varnothing )$ in *E* for some $p\in S$ and clock condition $\gamma$ that is true in *J*. In other words, $\Phi (S)$ comprises states reachable from the states of *S* by non-resetting transitions over *a* that are available for clock values in *J*. We define $\Psi (S)$ in a similar way, but for resetting transitions over *a* that are available for clock values in *J*.

First, we compute the output of the method, which is $\{(q,0)\ :\ q\in \Psi (S)\}$ where *S* is the set of all states appearing in the configurations of $L$. Note that, by Invariant (I4), *S* can be computed in time ${\mathcal {O}}(2^{|{\mathcal {A}}|})$ by iterating through the list ${\mathtt {r}}$ and computing the union of sets ${\mathtt {states}}(\beta )$ for roots $\beta$ appearing on it. Thus, the output can be computed in time ${\mathcal {O}}(2^{|{\mathcal {A}}|})$.

Second, we need to update the values of function $\lambda$ by applying all possible non-resetting transitions over *a*. This can be done by iterating through the list ${\mathtt {r}}$ and, for each root $\beta$ appearing on it, substituting ${\mathtt {states}}(\beta )$ with $\Phi ({\mathtt {states}}(\beta ))$. Note that since we assumed that for every state *q*, some transition over *a* is always available at *q*, it follows that $\Phi$ maps non-empty sets of states to non-empty sets of states. Hence, after this substitution the roots of ${\mathtt {f}}$ will still be assigned non-empty sets of states. However, Invariant (I5) may cease to hold, as some roots may now be assigned the same set of states.

We fix this as follows. For every root $\beta$ of ${\mathtt {f}}$, inspect the list ${\mathtt {r}}$ and find the root $\beta '$ that has the largest rank among those satisfying ${\mathtt {states}}(\beta )={\mathtt {states}}(\beta ')$. If $\beta =\beta '$, then do nothing. Otherwise, turn $\beta$ into a non-root node of ${\mathtt {f}}$, remove it from the list ${\mathtt {r}}$, set ${\mathtt {parent}}(\beta )=\beta '$, and increment ${\mathtt {\#children}}(\beta ')$ by one. Note that after applying this modification, the function $\lambda$ stored in the data structure stays the same, while Invariant (I5) becomes satisfied.

As for the other invariants, the satisfaction of Invariants (I2), (I3), and (I4) after the update is clear. However, we need to be careful about Invariant (I6), as we might have substantially modified the structure of the forest ${\mathtt {f}}$. Observe that each modification of ${\mathtt {f}}$ that we applied boils down to attaching a tree with a root of some rank *i* as a child of a tree with a root of some rank $j>i$. By Invariant (I6), the former tree has depth at most $i+1$, which is bounded from above by *j*. Thus, after the attachment, the depth of the latter tree cannot become larger than $j+1$. We conclude that Invariant (I6) is maintained as well.

Finally, note that since the number of roots of ${\mathtt {f}}$ is always bounded by $2^{|Q|}-1$, all the operations described above can be performed in time ${\mathcal {O}}(2^{|{\mathcal {A}}|})$.

## Lower Bound for Two-Clock Timed Automata with Additive Constraints

In this section, we prove a complexity lower bound for a variant of the dynamic acceptance problem. Ideally, we would like to prove that there is a timed automaton ${\mathcal {A}}$ with two clocks such that no data structure can support dynamic acceptance for ${\mathcal {A}}$ in time depending only on $|{\mathcal {A}}|$. This would imply that our result (Theorem 1) for the dynamic acceptance problem for single-clock timed automata cannot be generalised to the multiple-clock setting. We are not able to establish optimality in this sense. We can however prove a result along the same line, by considering timed automata extended with *additive constraints*, that is, having clock conditions of the form $\left( \sum _{\mathtt {x}\in Z} \mathtt {x}\right) \sim c$, and by relying on certain complexity assumptions (Conjecture 3, also known as 3SUM Conjecture).

The 3SUM Conjecture was introduced by Gajentaan and Overmars [27, 28] in a stronger form, which postulated the non-existence of *sub-quadratic* algorithms, that is, achieving running time $o(n^2)$. This formulation was refuted by Grønlund and Pettie [29], who gave an algorithm for 3SUM with running time ${\mathcal {O}}(n^{2}/(\log n / \log \log n)^{2/3})$ in the real RAM model, which can be improved to ${\mathcal {O}}(n^{2}(\log \log n)^2/\log n)$ when randomization is allowed. However, the existence of a strongly sub-quadratic algorithm is conjectured to be hard.

Recall that in the 3SUM problem, we are given a set *S* of positive real numbers and the question is to determine whether there exist $a,b,c\in S$ satisfying $a+b=c$. We remark that the original phrasing of the conjecture allows non-positive numbers on input and asks for $a,b,c\in S$ such that $a+b+c=0$. It is easy to reduce this standard formulation to our setting, for example by replacing *S* with $S'=\{3M+x \,:\, x\in S\}\cup \{6M-x \,:\, x\in S\}$, where *M* is any real that is larger than the absolute value of *a*, for all $a\in S$.

The 3SUM Conjecture has received significant attention in the recent years, as it was realised that it can be used as a base for tight complexity lower bounds for a variety of discrete graph problems, including questions about efficient dynamic data structures [1, 30–32]. In this setting, it is common to assume the integer formulation of the conjecture: * there exists* $d\in {\mathbb {N}}$ *such that the* 3SUM *problem, where the input numbers are integers from the range* $[-n^d,n^d]$, *cannot be solved in strongly sub-quadratic time, assuming the word RAM model with words of bit-length* ${\mathcal {O}}(\log n)$. The construction we are going to present in this section proves an analogous lower bound for the dynamic acceptance problem, assuming the former integer formulation of the 3SUM Conjecture. For this, we would need to amend the formulation of the dynamic acceptance problem, so that it makes sense to use the word RAM model instead of the real RAM model. More precisely, we assume that (1) the input stream is finite and expected to have total length at most *N*, (2) the clock constants and the time spans in the stream are integers of bit-length at most *M*, and (3) the data structure solving the monitoring problem should work in the word RAM model with words of bit-length ${\mathcal {O}}(M+\log N)$.

We now prove Theorem 2. That is, we provide a lower bound for the dynamic acceptance problem for two-clock timed automata with additive constraints under the 3SUM Conjecture.

Our approach is similar in spirit to the other lower bounds on dynamic problems, which we mentioned above [1, 30–32]. We first prove 3SUM-hardness of deciding acceptance by a timed automaton with additive constraints in the static setting. We then show that any data structure that supports monitoring in amortized strongly sub-linear time would violate the 3SUM-hardness of the former static acceptance problem, thus proving Theorem 2.

The Proof of Theorem 2 follows almost directly from an analogous 3SUM-hardness result in the static setting:

### Lemma 4

If the 3SUM Conjecture holds, then there is a two-clock timed automaton ${\mathcal {A}}$ with additive constraints for which there is no algorithm that, given a finite timed word $w\in (\Sigma \uplus {\mathbb {R}}_{> 0})^*$ as input, where $\Sigma$ is a two-letter alphabet, decides whether ${\mathcal {A}}$ accepts *w* in time ${\mathcal {O}}(n^{2-\delta })$ for any $\delta >0$ and for $n=|w|$.

### Proof

We construct a two-clock timed automaton ${\mathcal {A}}$ with additive constraints and an algorithm that, given a set *S* of *n* positive reals, outputs in time ${\mathcal {O}}(n \log n)$ a word $w\in (\Sigma \uplus {\mathbb {R}}_{> 0})^*$ such that *w* is accepted by ${\mathcal {A}}$ if and only if there are $a,b,c\in S$ satisfying $a+b=c$. We find it more convenient to first present the construction of *w* from *S*. Then we present the automaton ${\mathcal {A}}$ and analyse its runs on *w*.

Let $S=\{s_1,s_2,\ldots ,s_n\}$ be a set of positive real numbers and let $M=\max (S)+1$. By sorting *S* we may assume that $0<s_1<\ldots<s_n<M$. We set $\Sigma =\{\diamondsuit ,\spadesuit \}$. The word obtained from *S* is defined as

$$\begin{aligned} w = u\ \spadesuit \ u\ \spadesuit \ v, \end{aligned}$$

where

$$\begin{aligned} u= & {} 2(M-s_n)\ \diamondsuit \ 2(s_n-s_{n-1})\ \diamondsuit \ 2(s_{n-1}-s_{n-2})\ \diamondsuit \ \ldots \ \diamondsuit \ 2(s_2-s_1)\ \diamondsuit \ 2(s_1-0);\\ v= & {} \,(M-s_n)\ \diamondsuit \ \,(s_n-s_{n-1})\ \diamondsuit \ \,(s_{n-1}-s_{n-2})\ \diamondsuit \ \ldots \ \diamondsuit \ \,(s_2-s_1)\ \diamondsuit . \end{aligned}$$

Note that *w* has length ${\mathcal {O}}(n)$ and can be constructed from *S* in time ${\mathcal {O}}(n\log n)$. Intuitively, the factors *u*, *u*, and *v* above are responsible for the choice of *a*, *b*, and *c*, respectively.

**Fig. 5:**
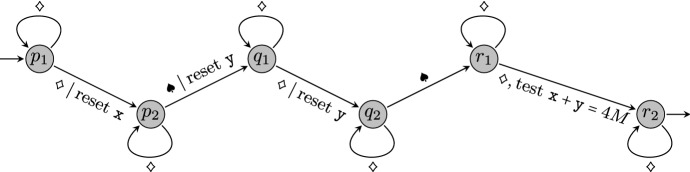
Timed automaton for reducing 3SUM

We now describe a timed automaton ${\mathcal {A}}$ that accepts *w* if and only if there are $a,b,c\in S$ such that $a+b=c$. The automaton is depicted in Fig. 5. It uses two clocks, named $\mathtt {x}$ and $\mathtt {y}$. All the transitions have trivial (always true) clock conditions, apart from the transition from $r_1$ to $r_2$, where we check that the sum of clock values is equal to 4*M*. The only initial state is $p_1$; the only accepting state is $r_2$.

Next, we analyse the runs of ${\mathcal {A}}$ on *w*, with the goal of showing that ${\mathcal {A}}$ accepts *w* if and only if there are $a,b,c\in S$ such that $a+b=c$. Consider any successful run $\rho$ of ${\mathcal {A}}$ on *w*. Observe that the moment of reading the first symbol $\spadesuit$ in *w* must coincide with firing the transition from $p_2$ to $q_1$. At this moment, the automaton has consumed the first factor *u* of *w*, and there was a moment where it moved from state $p_1$ to state $p_2$ upon reading one of the $\diamondsuit$ symbols from *u*. Supposing that the transition in $\rho$ from $p_1$ to $p_2$ happens at the *i*-th symbol $\diamondsuit$ of *u*, the clock valuation at the moment of reaching $q_1$ for the first time must satisfy $\mathtt {x} = 2(s_i-s_{i-1})+\ldots +2(s_2-s_1)+2s_1$ ($=2s_i$). We conclude the following.

### Claim 4.1

The set of possible valuations for clock $\mathtt {x}$ at the moment of reaching the state $q_1$ for the first time is $\{2a \,:\, a\in S\}$.

Next, observe that the moment of reading the second occurrence of $\spadesuit$ in *w* must coincide with firing the transition from $q_2$ to $r_1$. Between the first and the second symbol $\spadesuit$ the automaton consumes the second factor *u*, and the clock $\mathtt {x}$ increases exactly by the sum of the time spans within *u*, *i.e.*, by 2*M*. On consuming the second factor *u*, the clock $\mathtt {y}$ is reset once, and precisely when firing the transition from $q_1$ to $q_2$, which happens upon reading one of the occurrences of $\diamondsuit$ in *u*. Again, if this happens when reading the *j*-th occurrence of $\diamondsuit$, then, after the reset, $\mathtt {y}$ is incremented by exactly $2s_j$ units. We conclude the following.

### Claim 4.2

The set of possible clock valuations at the moment of reaching the state $r_1$ for the first time is $\{(\mathtt {x}=2a+2M, ~ \mathtt {y}=2b) \,:\, a,b\in S\}$.

Finally, after consuming the last factor *v*, the automaton can move to the accepting state $r_2$ if and only if at some point, upon reading an occurrence of $\diamondsuit$, the condition $x+y=4M$ holds. Observe that the sum of the first *k* numbers encoded in *v* is equal to $M-s_{n-k+1}$. Hence, after parsing those numbers, the set of possible clock valuations is $\{(\mathtt {x}=2a+2M+M-c, ~ \mathtt {y}=2b+M-c) \,:\, a,b\in S\}$, for some choice of $c\in S$. Moreover, the latter valuations satisfy the condition $\mathtt {x}+\mathtt {y}=4M$ if and only if $a+b=c$.

Based on the above arguments, we infer that a successful run like $\rho$ exists on input *w* if and only if there are $a,b,c\in S$ such that $a+b=c$. To conclude the proof, we observe that if an algorithm could decide whether ${\mathcal {A}}$ accepts *w* in time ${\mathcal {O}}(n^{2-\delta })$ for any $\delta >0$, then by combining this algorithm with the presented construction, one could solve 3SUM in time ${\mathcal {O}}(n^{2-\delta })$. This would contradict the 3SUM Conjecture. $\square$

Now, Theorem 2, which we restate below for convenience, follows almost directly from the previous lemma.

**Theorem** 2  *If the* 3SUM *Conjecture holds, then there is a two-clock timed automaton* ${\mathcal {A}}$ *with additive constraints such that there is no data structure that, when initialized on* ${\mathcal {A}}$, *supports dynamic acceptance in time* ${\mathcal {O}}(n^{1-\delta })$ *for any* $\delta >0$, *where* *n* *is the length of the consumed stream prefix.*

### Proof

Consider the timed automaton ${\mathcal {A}}$ provided by Lemma 4. If a data structure as in the statement of the theorem existed, then using this data structure one could decide in strongly sub-quadratic time whether any input timed word *w* is accepted by ${\mathcal {A}}$, by simply applying the sequence of $\mathtt {read}_{}{(\cdot )}$ operations corresponding to *w*, followed by the query ${\mathtt {accepted}}$. $\square$

## Concluding Remarks and Future Work

In this work we studied the dynamic acceptance problem for timed automata processing data streams. We designed a suitable data structure for one-clock timed automata, where the amortized update time depends only on the size of the automaton. We leave as an open question whether this result can be generalised to the case of multiple clocks.

More generally speaking, it seems that our work identifies dynamic variants of classic automata problems as a potential area of interest for the paradigm of parametrised dynamic data structures. More precisely, if the automaton model in question allows for the device to potentially be in an unbounded number of configurations, then the dynamic maintenance of this set of configurations is a computationally challenging problem, as show-cased in this paper. There are multiple models of devices where similar questions can be asked. Examples include counter automata, register automata, weighted automata, or pushdown automata.
